# Clinical Outcomes in Men and Women following Total Knee Arthroplasty with a High-Flex Knee: No Clinical Effect of Gender

**DOI:** 10.1155/2015/285919

**Published:** 2015-09-16

**Authors:** Jeffrey M. Nassif, William S. Pietrzak

**Affiliations:** ^1^Physicians' Clinic of Iowa, 202 10th Street Southeast, No. 140, Cedar Rapids, IA 52403, USA; ^2^Department of Bioengineering, University of Illinois at Chicago, 851 S. Morgan Street, Chicago, IL 60607, USA

## Abstract

While it is generally recognized that anatomical differences exist between the male and female knee, the literature generally refutes the clinical need for gender-specific total knee prostheses. It has been found that standard, unisex knees perform as well, or better, in women than men. Recently, high-flex knees have become available that mechanically accommodate increased flexion yet no studies have directly compared the outcomes of these devices in men and women to see if gender-based differences exist. We retrospectively compared the performance of the high-flex Vanguard knee (Biomet, Warsaw, IN) in 716 male and 1,069 female knees. Kaplan-Meier survivorship was 98.5% at 5.6–5.7 years for both genders. After 2 years, mean improvements in Knee Society Knee and Function scores for men and women (50.9 versus 46.3; 26.5 versus 23.1) and corresponding SF-12 Mental and Physical scores (0.2 versus 2.2; 13.7 versus 12.2) were similar with differences not clinically relevant. Postoperative motion gains as a function of preoperative motion level were virtually identical in men and women. This further confirms the suitability of unisex total knee prostheses for both men and women.

## 1. Introduction

Morphometric differences exist between the male and female knee populations. Such differences include smaller size [1, 2], larger Q-angle [1, 3, 4], smaller observable prominence of the anterior condyle [1], smaller medial-lateral (ML) to anterior-posterior (AP) femoral condyle aspect ratio [1–3, 5], and thinner patella [6] in female knees compared to male knees. This has led to the thought that standard total knee implants in women may have a tendency to overstuff the patellofemoral compartment leading to a reduced range of motion and increased overhang with subsequent lateral and medial knee pain due to soft tissue irritation [7]. Gender-specific knee designs attempt to address these concerns through design modifications to better accommodate the female femoral condyle such as modifying the anterior flange to include a recessed sulcus and reduced anterior condyle height, reducing the ML : AP aspect ratio, and increasing the angle of the trochlear groove [3, 7]. The most studied gender-specific knee system is the Zimmer Gender Solutions NexGen Knee (Zimmer Inc., Warsaw, IN). Such investigations have included unilateral [8, 9] and bilateral [9–12] studies in women as well as Indian [13] and Tai [14] patients. The literature, however, provides little support for gender-specific prostheses. Following a systematic review, Merchant et al. [15] concluded that the apparent anatomic differences between male and female knees were due to the smaller height and size of women and not due to gender, per se. Their review also indicated that rather than women having poorer total knee arthroplasty (TKA) results than men using a standard prosthesis, female outcomes are actually as good or better. A systematic review and meta-analysis by Xie et al. [7] also found no evidence to support the need for gender-specific knees. In addition to studies showing that outcomes are similar between the sexes with standard knees [15–19], it has also been shown that outcomes are not substantially different in women whether they receive a standard or gender-specific knee [8, 10–13].

Recently, there has been interest in high-flex knees [20–22], that is, those that can mechanically accommodate flexion in excess of 125° (ASTM F2083); however their ability to equally serve males and females has not been established. Only one small intraoperative study (40 patients) [9] and a few short-term small cohort (<50 patients) [10, 12] or intermediate cohort (up to 138 patients) [8, 11, 13] studies have compared a high-flex knee with a gender-specific knee, finding no clear advantage of the latter. The flexion achieved by a given patient will be dependent upon the amount that can be accommodated by the design of the knee and anatomical features of the patient including soft tissue restraint that is often a limiting factor. In the case of high-flex knees, the limiting effect of patient anatomy may be more evident than would be the case for standard knees.

The purpose of this study was to compare the mid-term functional outcomes and survivorship of large male and female cohorts receiving the same cruciate-retaining (CR) high-flex knee. Our hypothesis was that there would be no difference in these metrics between the genders with this prosthesis.

## 2. Materials and Methods

The Vanguard knee (Biomet, Warsaw, IN) is a high-flex design as it can mechanically accommodate up to 145° of flexion although that achieved clinically may be less due to the soft tissue restraint of the patient [20, 23]. From September 2004 to April 2013, a consecutive series of 1,328 patients received 1,785 cruciate-retaining (CR) knees of this type via a medial parapatellar approach using cemented fixation, including 802 women (1,069 knees) and 526 men (716 knees). All patellae were resurfaced. Standard overlay templating was used to determine proper implant size until 2011, after which digital templating was employed. There are ten nominal sizes of femoral components, that is, 55–75 in increments of 2.5, plus 80, and nine nominal tibial component sizes, that is, 59–91 in increments of 4 in this system. All femoral and tibial component sizes are compatible with each other throughout their respective ranges. The tibial component was sized for best fit on the cut surface without AP or ML overhang. The femoral component was sized using the posterior referencing femoral sizer that was part of the system. All surgeries were performed by the senior author at a single site following Institutional Review Board approval and signed consent from each patient. Mean follow-up for the male and female patients was 2.4 years (range: 0.8–6.9 years) and 2.4 years (range: 0.7–7.2 years), respectively.

### 2.1. Survivorship Analysis

Kaplan-Meier survivorship analysis was performed for each gender (716 male knees and 1,069 female knees), including 95% confidence limits, with the endpoint defined as revision of any component for any reason. Final survivorship intervals were chosen to correspond to those at which 20 knees remained at risk to avoid the instability that can result when the remaining population becomes too small [24]. This final interval was 5.6 years for men and 5.7 years for women.

### 2.2. Functional Analysis

Functional analysis was performed on only those knees with a minimum of 2 years of complete clinical follow-up. Clinical assessment consisted of preop and final postop Knee Society Score (KSS) [25] and SF-12 [26]. Three hundred thirteen male patients (397 knees) and 463 female patients (574 knees) had complete preop and minimum 2-year postop clinical assessment, with mean final follow-up of 2.9 years (range: 2.0–6.9 years) and 2.9 years (range: 2.0–7.2 years), respectively.

Preoperative and ≥2-year postoperative passive range of motion (ROM) and passive peak flexion (PF) data was available for 462 male and 671 female knees. Motion results were stratified by preoperative motion range, that is, <95°, 95–105°, and >105°.

### 2.3. Statistical Analysis

Interval data means between the genders (patient age, body mass index, length-of-stay, length of follow-up, KSS, SF12, ROM, and PF) were compared using the pooled *t*-test. Preop to postop changes in outcomes for a given gender were compared by the paired *t*-test. Nominal data mean differences (proportion of right knees and distribution of primary diagnoses) were compared by the Chi-square test. A value of *p* < 0.05 was chosen for statistical significance.

## 3. Results 

Table 1 summarizes the patient demographics for the total cohorts as well as those limited to a minimum of 2-year complete follow-up. No significant gender difference was seen in primary diagnosis (*p* = 0.409), proportion of right knees (*p* = 0.271), or length of follow-up (*p* = 1.0). However, the differences in patient age (*p* = 0.032), body mass index (*p* = 0.009), and length-of-stay (*p* < 0.001) for males compared to females were significant. A total of 37 patients died for reasons unrelated to their knee procedure (13 men with 18 knees and 15 women with 19 knees) with all implants in place at the time of death.

Table 2 presents the KSS and SF-12 results. Preoperatively, men and women had similar SF-12 Physical scores while women had greater KS Knee scores and men had greater KS Function and SF-12 Mental scores. Postoperatively, men and women had similar KS Knee scores, with men having greater KS Function and SF-12 (both components) scores. For both men and women there was a significant preop to postop increase (*p* < 0.0001) in both components of the KSS and the SF-12, with the exception of the SF-12 Mental score for men.

Table 3 lists the means of the preop to postop differences (Δscores) for the men and women. While the gender differences were not pronounced, they did reach statistical significance, with greater KS Knee and Function and SF-12 Physical Δscores for men and greater SF-12 Mental Δscore for women.

Tables 4 and 5 stratify the male and female ROM and PF results, respectively, by preop motion with two principal observations apparent. First, the improvement in motion (ΔROM and ΔPF) was inversely related to the preop motion of the patient, that is, knees with less motion prior to surgery tended to achieve a greater increase after surgery than did knees initially presenting with a high degree of motion. Second, there were no significant differences in the pre- to postop change in motion between men and women with the exception of a 2-degree PF differential in favor of men for the mid-functioning preop group. The same proportion of male and female knees (81–83%) achieved ≥120° of ROM and PF with no significant differences between the genders (ROM: *p* = 0.605, PF: *p* = 0.423).

A total of seven revisions were performed including four male knees at 0.65 years (aseptic loosening), 0.73 years (infection), 2.01 years (infection), and 2.13 years (aseptic loosening), and three female knees at 2.51 years (infection), 4.18 years (aseptic loosening), and 5.25 years (dislocation). All components were replaced in four revisions, only the tibial component and liner in two revisions, and the femoral component, tibial component, and liner in one revision. Kaplan-Meier survivorship was identical in both cohorts, that is, 98.5% (95% CI: 97.8–99.2%) at 5.6 years for males and 98.5% (95% CI: 96.5–100%) at 5.7 years for females.

## 4. Discussion

There is little doubt that morphometric differences exist between female and male knees [1–6, 27]. This has led to the hypothesis that standard (unisex) knees designed without regard to gender differences could produce inferior outcomes in women and that a gender-specific knee would be required to address this issue [3, 7]. Systematic reviews and meta-analysis of the literature, however, suggest the opposite; that is, women obtain equivalent, if not better, outcomes than men using standard knees [7, 15]. Other studies that collectively compared 8,700 female knees with 5,927 male knees, both receiving standard implants, came to the same conclusion [16–19]. In recent years, high-flex knees have been developed to provide increased flexion potential which is especially useful for high-demand patients [20–22]. It is possible that gender-related differences in outcomes might become manifest with the use of such high-performance prostheses since such devices could potentially intensify the influence of knee morphology on clinical performance.

Four studies collectively examined 308 bilateral female patients, each having one knee replaced with a gender-specific high-flex knee and the other knee receiving a unisex knee (high-flex or non-high-flex) [10–13]. While there was no apparent advantage of the gender-specific high-flex design in women, these studies did not directly address the need for gender-specific high-flex knees since no male patients were included with which to compare outcomes. Ours was the first study to compare the use of a unisex high-flex knee in both male and female cohorts. The high-flex CR knee design used in this study was chosen to address this issue, in part, because there have been recent large-cohort, mid-term studies published on this system to provide a baseline to which our results may be compared [20, 28].

In our study, the total population of 1,785 high-flex cruciate-retaining knees resulted in only seven revisions. Stratifying survivorship by gender yielded Kaplan-Meier survivorship estimates of 98.5% for both men and women at 5.6 years and 5.7 years, respectively. Other investigators have found similar Kaplan-Meier survivorship for the same knee, that is, 97.8% at 7.0 years reported by Schroer et al. [20] (957 knees; 85.0% posterior-stabilized, 15.0% cruciate-retaining; 36.5% male; 63.5% female) and 98.6% at 6.0 years reported by Kievit et al. [28] (807 knees; 51.3% posterior-stabilized, 48.7% cruciate-retaining; 35.8% male, 64.2% female).

With the exception of the KS Knee score, the preoperative condition of the male knees was better than that of the female knees, which was significant (*p* < 0.01) for all but the physical component of the SF-12. Others have reported similar findings likely because women tend to present later for surgery, with lower function and pain scores than men [16, 17, 19, 29]. Both men and women showed significant increases (*p* < 0.0001) in both components of the KSS and SF-12 with the exception of the SF-12 Mental component for males. Schroer et al. [20] observed similar improvement in KSS for a combined male-female population receiving this knee.

As regards the KSS and SF-12 outcome comparisons between the men and women, it is best to compare the Δscores since the preop scores were different. The Δscores were significantly greater for males than females for KS Knee score (50.9 versus 46.3, *p* = 0.002), KS Function score (26.5 versus 23.1, *p* = 0.026), and the SF-12 Physical score (13.7 versus 12.2, *p* = 0.031), while females were ahead with the SF-12 Mental score (2.2 versus 0.2, *p* = 0.003). Consequently, the statistical comparisons of the score improvements did not show a consistent advantage for either gender.

In general, the change in motion following TKA is inversely related to the preoperative value, that is, patients presenting with restricted motion tend to gain much motion after surgery while those with a high degree of motion initially tend to stay about the same, or perhaps lose a small amount of motion, after surgery [20, 23]. While this “regression toward the mean” is well documented, a potential gender effect has not been previously investigated. When stratifying motion outcomes by their preop values, that is, <95°, 95°–105°, and >105°, we found virtually identical ΔROM and ΔPF values for both men and women, that is, ΔROM: 32°–34°, 18°, and 4° and ΔPF: 23°–24°, 15°–17°, and 4°, respectively. As such, no gender influence on motion was apparent in general or as a function of preop motion. By way of comparison, Schroer at al. [20] documented similar Δ PF in a combined male-female population (two-thirds female) receiving the same knee (627 knees; 509 posterior-stabilized and 118 cruciate-retaining) of 23.6°, 19.3°, and 1.8° in these three preop categories, respectively.

Taken in aggregate, the survivorship, KSS, SF-12, ROM, and PF values suggest similar performance, overall, of this CR high-flex knee in both men and women. While there were some statistically significant differences in some outcomes between the genders, these differences were small and were of the magnitude obtained by others who did not ascribe clinical relevancy to them [10–13, 16–19].

Gender considerations notwithstanding, current trends in high-flex knee design, include reducing the posterior radius of curvature which increases the contact area between the posterior femoral condyle and the tibial insert [30]. This increase in contact area, however, may not be sufficient to effectively distribute the high forces developed during deep squatting which can potentially lead to increased polyethylene wear. Extreme flexion may increase patellofemoral joint stress and disrupt patellar-trochlear groove congruity, leading to other complications such as pain, patellar fracture, and patellar loosening [30]. In our study of 1,785 high-flex knees, a total of seven (0.39%) revisions were performed, including three (0.17%) for infection, three (0.17%) for aseptic loosening, and one (0.06%) for dislocation. As such, no particular sequelae associated with the high-flex knee design were evident.

So what does this all mean? First, the literature has generally concluded that standard, unisex knee designs are equally suitable for both men and women. Second, our study helped fill a void in the literature by comparing the same unisex high-flex knee design in both men and women, thereby extending the results of others while reaching the same conclusions. Third, the complete compatibility of the entire range of femoral and tibial component sizes of the studied knee with each other may have allowed sufficient latitude to address patient needs regardless of gender. In other words, knee gender differences can be largely addressed through implant size rather than implant design considerations.

There were limitations in our study that should be considered. First, this was a retrospective study so there may be inherent biases that could have influenced the results. Despite this, the knee populations were large which may have partially mitigated this limitation. Second, only one type of high-flex knee was studied. As such, these results cannot be directly extended to other high-flex knee designs. Third, only mid-term survivorships were reported. Long-term survivorships of at least 10 years will be required to fully document gender-related outcomes differences that may exist with this knee design.

In summary, we found this knee to be highly effective in both men and women as evidenced by significant improvement in KSS and SF-12, similar ROM and PF outcomes, and high mid-term survivorship of 98.5%, confirming our study hypothesis.

## 5. Conclusion

It is important that surgeons have the necessary information available to make an informed decision about treatment options for their patients. This is particularly true in joint replacement where there are a plethora of implant types and design philosophies. The contention that women have inferior outcomes following standard TKA, which is the* raison d'être* for gender-specific designs, has been dispelled by the literature. We have further solidified and extended this position by showing that one particular high-flex knee design had comparable clinical effectiveness in men and women.

## Figures and Tables

**Table 1:** Patient demographics.

Parameter	Total		With ≥ 2-year complete follow-up	
	Female	Male	Female	Male
Number of patients	802	526	463	313
Number of knees	1069	716	574	397
Proportion right knees	51.7%	49.4%	51.4%	49.4%
Patient age, mean (range) years	71.9 (39–95)	70.9 (44–96)	73.3 (50–94)	71.6 (48–95)
Body mass index mean (range) kg/m^2^	33.2 (18–76)	32.3 (8–79)	32.6 (18–62)	32.0 (8–57)
Diagnosis				
Osteoarthritis	97.5%	98.6%	97.4%	98.7%
Rheumatoid arthritis	1.6%	0.8%	1.9%	1.0%
Avascular necrosis	0.6%	0.3%	0.4%	0.3%
Osteonecrosis	0.1%	0.0%	0.2%	0.0%
Posttraumatic arthritis	0.1%	0.1%	0.2%	0.0%
Others	0.2%	0.1%	0.0%	0.0%
Length-of-stay, mean (range) days	2.5 (1–18)	2.2 (1–22)	2.4 (1–10)	2.1 (1–11)
Follow-up, mean (range) years	2.4 (0.7–7.2)	2.4 (0.8–6.9)	2.9 (2–7.2)	2.9 (2–6.9)

**Table 2:** Knee Society Score and SF-12 outcomes summaries after a minimum of 2 years.

Gender	Score	Component	Preop, mean (range)	Postop, mean (range)	*p* value
Female	Knee Society Score	Knee	43.5 (0–100)^a^	89.8 (25–100)^e^	<0.0001
		Function	51.4 (0–100)^b^	74.5 (0–100)^f^	<0.0001
	SF-12	Physical	31.6 (12.5–56.7)^c^	43.8 (8.4–63.0)^g^	<0.0001
		Mental	51.8 (13.6–77.8)^d^	54.0 (26.3–70.6)^h^	<0.0001

Male	Knee Society Score	Knee	40.2 (0–93)^a^	91.0 (32–100)^e^	<0.0001
		Function	59.8 (5–100)^b^	86.3 (20–100)^f^	<0.0001
	SF-12	Physical	32.4 (12.1–56.7)^c^	46.0 (14.3–65.0)^g^	<0.0001
		Mental	55.5 (21.2–75.5)^d^	55.7 (31.4–72.5)^h^	0.729

*p* values for intra-preop and intra-postop comparisons: a = 0.0081, b = <0.0001, c = 0.141, d = <0.0001, e = 0.143, f = <0.0001, g = 0.0005, and h = 0.0014.

**Table 3:** Comparison of preop to postop score changes (Δscores).

Score	Δscores (average ± SD)		*p* value
	Female (*n* = 574)	Male (*n* = 397)	
KSS Knee	46.3 ± 22.6	50.9 ± 21.8	0.002
KSS Function	23.1 ± 25.2	26.5 ± 20.2	0.026
SF-12 Mental	2.2 ± 10.3	0.2 ± 10.1	0.003
SF-12 Physical	12.2 ± 10.7	13.7 ± 10.5	0.031

**Table 4:** Range of motion (ROM) comparisons after a minimum of 2 years.

Preop ROM	Female		Male		*p* value for male versus female ΔROM^b^
	Postop ROM (°)^a^	ΔROM (°)^a,b^	Postop ROM (°)^a^	ΔROM (°)^a,b^	
<95°	113.2 ± 10.0 (*n* = 76)	34.1 ± 11.0 (*n* = 76)	117.4 ± 8.4 (*n* = 51)	32.3 ± 8.7 (*n* = 51)	0.329
95°–105°	118.9 ± 4.5 (*n* = 204)	17.5 ± 5.8 (*n* = 204)	119.3 ± 4.5 (*n* = 133)	17.9 ± 6.0 (*n* = 133)	0.542
>105°	119.9 ± 5.4 (*n* = 391)	3.6 ± 6.7 (*n* = 391)	120.3 ± 5.8 (*n* = 278)	4.2 ± 8.0 (*n* = 278)	0.293

Note: ^a^mean ± SD; ^b^ΔROM is the paired difference between preop and postop ROM.

**Table 5:** Peak flexion (PF) comparisons after a minimum of 2 years.

Preop flex	Female		Male		*p* value for male versus female ΔPF^b^
	Postop PF (°)^a^	ΔPF (°)^a,b^	Postop PF (°)^a^	ΔPF (°)^a,b^	
<95°	111.4 ± 13.2 (*n* = 28)	23.9 ± 12.1 (*n* = 28)	110.9 ± 12.2 (*n* = 11)	23.2 ± 10.3 (*n* = 11)	0.867
95°–105°	117.1 ± 5.6 (*n* = 73)	15.2 ± 5.7 (*n* = 73)	119.0 ± 4.5 (*n* = 44)	17.4 ± 5.7 (*n* = 44)	0.045^c^
>105°	119.9 ± 4.7 (*n* = 570)	3.9 ± 6.2 (*n* = 570)	120.2 ± 4.7 (*n* = 407)	3.9 ± 6.6 (*n* = 407)	1.000

Note: ^a^mean ± SD; ^b^ΔPF is the paired difference between preop and postop PF; ^c^significant.

## References

[1] Dargel J., Michael J. W. P., Feiser J., Ivo R., Koebke J. (2011). Anatomy revisited: morphometry in the light of sex-specific total knee arthroplasty. *Journal of Arthroplasty*.

[2] Greene K. A. (2007). Gender-specific design in total knee arthroplasty. *Journal of Arthroplasty*.

[3] Conley S., Rosenberg A., Crowninshield R. (2007). The female knee: anatomic variations. *Journal of the American Academy of Orthopaedic Surgeons*.

[4] Woodland L. H., Francis R. S. (1992). Parameters and comparisons of the quadriceps angle of college-aged men and women in the supine and standing positions. *American Journal of Sports Medicine*.

[5] Guy S. P., Farndon M. A., Sidhom S., Al-Lami M., Bennett C., London N. J. (2012). Gender differences in distal femoral morphology and the role of gender specific implants in total knee replacement: a prospective clinical study. *Knee*.

[6] Hitt K., Shurman J. R., Greene K. (2003). Anthropometric measurements of the human knee: correlation to the sizing of current knee arthroplasty systems. *The Journal of Bone & Joint Surgery—American Volume*.

[7] Xie X., Lin L., Zhu B., Lu Y., Lin Z., Li Q. (2013). Will gender-specific total knee arthroplasty be a better choice for women? A systematic review and meta-analysis. *European Journal of Orthopaedic Surgery and Traumatology*.

[8] Von Roth P., Matziolis G., Pfitzner T. (2013). Early results of gender-specific posterior stabilized total knee arthroplasty without patella resurfacing. *Orthopade*.

[9] Song E. K., Park S. J., Yoon T. R., Park K. S., Seo H. Y., Seon J. K. (2012). Hi-flexion and gender-specific designs fail to provide significant increases in range of motion during cruciate-retaining total knee arthroplasty. *Journal of Arthroplasty*.

[10] Thomsen M. G., Husted H., Bencke J., Curtis D., Holm G., Troelsen A. (2012). Do we need a gender-specific total knee replacement? A randomised controlled trial comparing a high-flex and a gender-specific posterior design. *Journal of Bone & Joint Surgery—British Volume*.

[11] Kim Y.-H., Choi Y., Kim J.-S. (2010). Comparison of standard and gender-specific posterior-cruciate-retaining high-flexion total knee replacements: a prospective, randomized study. *Journal of Bone and Joint Surgery B*.

[12] Song E. K., Jung W. B., Yoon T. R., Park K. S., Seo H. Y., Seon J. K. (2012). Comparison of outcomes after bilateral simultaneous total knee arthroplasty using gender-specific and unisex knees. *Journal of Arthroplasty*.

[13] Singh H., Mittal V., Nadkarni B., Agarwal S., Gulati D. (2012). Gender-specific high-flexion knee prosthesis in Indian women: a prospective randomised study. *Journal of Orthopaedic Surgery*.

[14] Tanavalee A., Rojpornpradit T., Khumrak S., Ngarmukos S. (2011). The early results of gender-specific total knee arthroplasty in Thai patients. *Knee*.

[15] Merchant A. C., Arendt E. A., Dye S. F. (2008). The female knee: anatomic variations and the female-specific total knee design. *Clinical Orthopaedics and Related Research*.

[16] Dalury D. F., Mason J. B., Murphy J. A., Adams M. J. (2009). Analysis of the outcome in male and female patients using a unisex total knee replacement system. *The Journal of Bone and Joint Surgery—British Volume*.

[17] MacDonald S. J., Charron K. D., Bourne R. B., Naudie D. D., McCalden R. W., Rorabeck C. H. (2008). The John Insall award: gender-specific total knee replacement: prospectively collected clinical outcomes. *Clinical Orthopaedics and Related Research*.

[18] Muenzberg M., Stretz C., Baur W., Stangl R., Merschin D. (2014). Gender influence on the outcome of an unisex total knee arthroplasty system. *Technology and Health Care*.

[19] Ritter M. A., Wing J. T., Berend M. E., Davis K. E., Meding J. B. (2008). The clinical effect of gender on outcome of total knee arthroplasty. *Journal of Arthroplasty*.

[20] Schroer W. C., Stormont D. M., Pietrzak W. S. (2014). Seven-year survivorship and functional outcomes of the high-flexion Vanguard complete knee system. *The Journal of Arthroplasty*.

[21] Endres S., Wilke A. (2011). High flexion total knee arthroplasty—mid-term follow up of 5 years. *The Open Orthopaedics Journal*.

[22] Cho S.-D., Youm Y.-S., Park K.-B. (2011). Three- to six-year follow-up results after high-flexion total knee arthroplasty: can we allow passive deep knee bending?. *Knee Surgery, Sports Traumatology, Arthroscopy*.

[23] Stormont D. M., Chillag K. J., Scott J. W., Klaassen M. A., Pietrzak W. S. (2009). The relationship between pre- and postoperative range of motion utilizing three cruciate-retaining total knee prostheses. *Journal of Investigative Surgery*.

[24] Dorey F., Amstutz H. C. (1986). Survivorship analysis in the evaluation of joint replacement. *Journal of Arthroplasty*.

[25] Insall J. N., Dorr L. D., Scott R. D., Scott W. N. (1989). Rationale of the knee society clinical rating system. *Clinical Orthopaedics and Related Research*.

[26] Jenkinson C., Layte R., Jenkinson D. (1997). A shorter form health survey: can the SF-12 replicate results from the SF-36 in longitudinal studies?. *Journal of Public Health Medicine*.

[27] Chin K. R., Dalury D. F., Zurakowski D., Scott R. D. (2002). Intraoperative measurements of male and female distal femurs during primary total knee arthroplasty. *The Journal of Knee Surgery*.

[28] Kievit A. J., Schafroth M. U., Blankevoort L., Sierevelt I. N., van Dijk C. N., van Geenen R. C. I. (2014). Early experience with the Vanguard complete total knee system: 2–7 years of follow-up and risk factors for revision. *The Journal of Arthroplasty*.

[29] Liebs T. R., Herzberg W., Roth-Kroeger A. M., Rüther W., Hassenpflug J. (2011). Women recover faster than men after standard knee arthroplasty. *Clinical Orthopaedics and Related Research*.

[30] Ranawat C. S. (2003). Design may be counterproductive for optimizing flexion after TKR. *Clinical Orthopaedics and Related Research*.

